# Treatment of Femoral Artery Thrombosis with Streptokinase and Heparin After Cardiac Catheterization

**DOI:** 10.5812/cardiovascmed.13552

**Published:** 2014-02-24

**Authors:** Hojjat Mortezaiyan, Mohammadyosef Aarabi-Moghadam, Nabiollah Asadpour, Sepideh Parchami-Ghazaee, Yasaman Khalili, Kourosh Vahidshahi

**Affiliations:** 1 Cardiovascular Intervention Research Center, Rajaei Cardiovascular Medical and Research Center, Iran University of Medical Sciences, Tehran, IR Iran; 2 Hajar Pediatric Medical and Research Center, Shahrekord, IR Iran; 3 Department of Community Medicine, Tehran University of Medical Sciences, Tehran, IR Iran

**Keywords:** Cardiac Catheterization, Thrombosis, Streptokinase and Thrombolysis

## Abstract

**Background:**

Thrombosis is the most common complication during cardiac catheterization via femoral artery access. Alongside heparinization, fibrinolytic therapy is recommended if there are signs of ischemia in the lower extremity.

**Objectives:**

Given the paucity of data in the existing literature on streptokinase (SK) therapy in pediatrics, we designed this study to assess the efficacy of SK in pediatric patients with diagnosed femoral artery thrombosis following cardiac catheterization.

**Patients and Methods:**

The study population initially consisted of 1788 pediatric patients who underwent cardiac catheterization via the femoral artery access. Diminished or absent pulses in the lower extremity were detected in 123 patients, 45 of whom (2.5% of 1788) required treatment and were therefore considered for the next stage of study. Treatment was comprised of post-procedural intravenous heparin, either 50 U/kg/Q4h or 10 - 20 U/kg/h continuously. After heparinization for 24 hours, if the pulse of the affected extremity was not palpable, heparin therapy was continued (heparin-treated group, n = 28), and if the symptoms of femoral artery ischemia were persistent, heparin was discontinued and intravenous SK with a loading dose of 2000 U/kg over 20 - 30 minutes was commenced (SK-treated group, n =17).

**Results:**

In the presence of pulselessness in the lower extremity, a maintenance dose of SK (1000 U/kg/h, during 1 - 24 hours) was intravenously administered. Regarding the return of the pulses post-therapeutically, normal and weak/absent pulses were detected in seven (25.2%) and 21 (74.8%) of the 28 patients, respectively, in the heparin-treated group (P value < 0.001), whereas normal and weak/absent pulses were detected in 15 (88.2%) and two (11.8%) of the 17 patients, respectively, in the SK-treated group (P value < 0.001).

**Conclusions:**

Our findings demonstrated a high success rate and a low complication rate for systemic SK therapy in femoral artery thrombosis after catheterization.

## 1. Background

Cardiac catheterization is an important diagnostic and therapeutic procedure in pediatric patients (1). However, it is still not free of complications, despite all the considerable improvements in technology and equipment, during the last decade (2). The femoral artery access during cardiac catheterization may be accompanied by complications such as bleeding, hematoma, arteriovenous fistula, and the most common of all, thrombosis (3, 4). Femoral artery ischemia is allied to pulselessness, cold temperature, pallor, pain and impaired movement of the lower extremity and, in more severe cases, may cause necrosis and obligatory amputation on the short term and claudication or impaired limb longitudinal growth on the long-term (5, 6). There are several methods to manage post-catheterization femoral artery thrombosis, including surgical thrombectomy, balloon dilatation and surgical excision of the thrombosis. Nevertheless, these modalities may be difficult and complicated in pediatric patients, especially in infants and children (7). Systemic heparinization reduces the risk of arterial thrombosis in pediatric patients, (8) and fibrinolytic therapy is recommended if the signs of ischemia in the lower extremity are persistent (9). Systemic fibrinolytic agents such as streptokinase (SK), urokinase and tissue-type plasminogen activator (TPA) can be drawn upon as alternative treatments for the management of femoral artery thrombosis. The SK is deemed more advantageous to TPA and urokinase, which are relatively expensive.

## 2. Objectives

There is currently a dearth of specific studies and evidence regarding SK therapy in pediatrics, especially in infants and young children. The present study was designed to evaluate the efficacy of SK therapy in pediatric patients with diagnosed femoral artery thrombosis after cardiac catheterization.

## 3. Patients and Methods

This double-blind clinical trial included 1788 pediatric patients who underwent cardiac catheterization through femoral artery access, with standard cardiac catheterization technology, at Rajaie Cardiovascular, Medical and Research Center, Tehran between May 2011 and February 2012. The study protocol was approved by the institutional Review Board and Ethics Committee of Tehran University of Medical Sciences. All participants initially received an intravenous bolus of heparin sulfate (Pharmaceutical Co., Iran, 50 - 100 U/kg) during diagnostic or interventional catheterization. A pediatric interventionist performed regular physical examinations of the patients' lower extremities regarding their vascular condition. The patients underwent Doppler ultrasonography (Sonoline G40, Siemens Medical Solution the USA, Mountain View, CA 94043, the USA) while the manifestations of arterial ischemia (pulse weakness/absence, coolness, pallor, pain, paresthesia, paresis, and marked impaired pedal movement) were under close observation.

The inclusion criteria comprised the diagnosis femoral artery thrombosis and cut-off or monophasic flow in the external iliac, common femoral artery or superficial femoral artery, according to physical examination, pulse oximetry monitoring or Doppler ultrasonography, despite continued heparinization for 24 hours. The exclusion criteria were any evidence of dissection, arterial spasm, absence of thrombosis or limited thrombosis in the deep femoral artery (detected by Doppler ultrasound), any history of active gastrointestinal or internal bleeding of a 2 - 4 week duration prior to the procedure, any history of central nervous system (CNS) bleeding or other CNS disorders, and traumatic or cardiopulmonary resuscitation lasting more than 10 minutes.

Diminished or absent pulses in the lower extremity were detected in 123 patients, 45 of whom (2.5% of 1788) required treatment and were, therefore, considered for the next stage of the study after obtaining an informed written consent from their parents or custodians. Treatment consisted of post-procedural intravenous heparin, either 50 U/kg q4h or 10 - 20 U/kg/h continuously. Partial thromboplastin time (PTT) was measured during heparin therapy and heparinization was continued unless PTT was greater than 90 seconds. After heparinization for 24 hours, if the pulse of the affected extremity was not palpable, heparin therapy was continued (heparin-treated group, n = 28), and if the symptoms of femoral artery ischemia were persistent, heparin was discontinued and intravenous SK (Streptase®, CSL Behring GmbH 35041, Marburg, Germany) with a bolus loading dose of 2000 U/kg over 20 - 30 minutes was commenced (SK-treated group, n = 17).

In the absence of pulse in the lower extremity and the absence of any SK complications such as bleeding (at the puncture site, mucosa, stomach, intestines, ears, nose, throat, and CNS), skin allergy, anaphylaxis, cardiac dysrhythmia, seizure, and paresis, a maintenance dose of SK (1000 U/kg/h, during 1 - 24 hours) was intravenously administered using infusion syringe pumps (Atom Syringe Pump, SN-123, Atom Medical, Japan). Lower extremity pulses and oximetry curve were assessed during and after the treatment. After pulse and saturation curves normalization, SK was discontinued. In the presence of coagulopathy (prothrombin time [PT] > 15 seconds, PTT > 60 seconds, and international normalized ratio [INR] > 1.5), fresh frozen plasma (10 cc/kg), and a single dose of hydrocortisone (Exir Co., Iran, 5 mg/kg) were intravenously prescribed before SK administration. If any complication associated with SK was observed, the infusion was discontinued and appropriate diagnostic/therapeutic management was performed. An expert pediatric interventionist monitored all the patients during the SK infusion.

In both groups, complete blood count with differential (CBC-diff.), platelets (Plt), PTT, and INR were determined before the thrombolytic treatment. The PTT, CBC-diff., and INR were measured each 12 hours during the infusion. Doppler ultrasound was reviewed 24 - 72 hours after the thrombolytic initiation. If there was pulse weakness/absence or coolness of the lower extremity despite thrombolytic treatment, the patients were treated with low molecular weight heparin (LMWH) (Clexane) for 7 - 10 days. To assess the thrombosis reconstruction, Doppler ultrasound was reviewed 2 weeks after hospital discharge. Twelve months follow-up for the patients revealed no future need for a surgical intervention. Statistical analysis was performed by using SPSS version 16.0 for Windows (SPSS Inc., Chicago, Illinois), employing appropriate statistical tests: the chi-squared test for the comparison of the qualitative variables and the independent t-test and the Mann-Whitney test for the comparison of the quantitative variables. A P < 0.05 was considered significant.

## 4. Results

Of the 1788 pediatric patients, undergone cardiac catheterization, 123 (6.7%) patients were detected with diminished or absent pulses in the lower extremity. Of these 123 patients, 78 were excluded because of pulse normalization in the affected artery after heparin administration (50 U/kg/Q6h or 10 - 20 U/kg/h continuously) or negative reports of femoral artery thrombosis by Doppler ultrasound, leaving a total of 45 patients (2.5% of 1788) for the next stage of study. The study population consisted of 26 males and 19 females, with a mean age of 17 ± 28 months (ranging from 1 to 144 months, 33 (73.3%) < 6 months old) and a mean body weight of 7.8 ± 4.5 kg (range = 3 - 28 kg). In 9 (42.2%) of the patients, cardiac catheterization was interventional and 4 (9.3%) of the patients reported a history of previous cardiac catheterization. With respect to the manifestations of ischemia, pallor, mottling, cooling in the lower extremity, and impaired pedal movement were observed in 3 (6.7%), 12 (26.7%), 34 (75.6%) and 4 (8/9%) of the heparin/SK-treated patients, respectively. Table 1 depicts the results of the Doppler ultrasound of the lower extremity in the heparin/SK-treated patients. The demographic and procedural characteristics of the heparin/SK-treated subjects are presented in Table 2. 

**Table 1.** Prevalence of Thrombosis and Blood Flow Cut-Off in Different Arteries Accessed in Heparin/SK-treated Patients (n = 45)

Accessed artery	Patients, No. (%)
**Superficial femoral**	16 (35.6)
**Common femoral**	10 (22.2)
**Superficial femoral and distal arteries**	4 (8.9)
**External iliac**	4 (8.9)
**Superficial and external iliac**	10 (22.2)
**Radial**	1 (2.2)

**Table 2.** Demographic and Catheterization Characteristics of Heparin/SK-treated Patients

Characteristics	Heparin-treated, n = 28	SK-treated ^a^, n = 17	P value
**Age, Mean ± SD**	17.01 ± 28	17.18 ± 29	0.985
**Gender, No. (%)**			0.051
Male	19 (67.9)	7 (41.2)	
Female	9 (32.1)	10 (58.8)	
**Angiography**			0.876
Diagnostic	15 (53.6)	10 (58.8)	
Interventional	13 (46.4)	7 (41.2)	
**History of angiography**	3 (10.7)	1 (5.9)	0.521
**Thrombus site**			0.271
Right leg	18 (64.2)	10 (58.8)	
Left leg	5 (17.9)	4 (23.5)	
Both	5 (17.9)	3 (20.7)	
**Vascular accesses**			0.144
Arterial	10(35.7)	7 (50)	
Venous	8 (28.6)	4 (23.5)	
Both	10(35.7)	6 (42.9)	
**Abnormal pulse oximetry**			0.407
Right foot	23 (82.1)	14 (83.4)	
Left foot	5 (17.9)	3 (17.6)	
**Manifestations of ischemia**			
Mottling	4 (14.3)	8 (47.1)	0.009
Pallor	1 (3.6)	2 (11.8)	0.009
Cooling	21 (75)	13 (76.5)	0.513
Impaired pedal movement	0	4 (23.5)	0.017
**Capillary returning time ^b^, s**			0.076
< 2	3 (15)	1 (9.1)	
2 - 5	17 (85)	7 (63.6)	
> 5	0	3 (27.3)	

^a^ Abbreviation: SK, streptokinase.

^b^ Eight records in group heparin and six records in group SK was missed.

**Figure 1.**
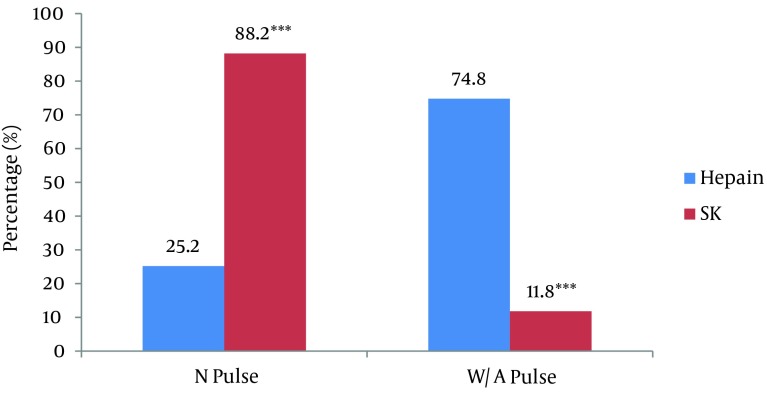
Prevalence of Normal or Weak/Absent Pulses Detected in Heparin/SK-Treated Patients after Treatment N Pulse, normal pulse; W/A Pulse, weak/absent pulse; SK, streptokinase; *** P value < 0.001.

The SK-treated group showed more severe signs of arterial ischemia in the lower extremity for pallor (P value = 0.009), mottling (P value = 0.009), and impaired pedal movement (P value = 0.017), but there were no statistically significant differences in other characteristics between the two groups. Figure 1 illustrates data on pulse return after therapy. Normal and weak/absent pulses were detected in seven (25.2%) and 21 (74.8%) of the 28 patients, respectively, in the heparin-treated group (P value < 0.001), whereas normal and diminished/absent pulses were detected in 15 (88.2%) and two (11.8%) of the 17 patients, respectively, in the SK-treated group (P value < 0.001).

The time of pulse return was significantly shorter in the SK-treated group (12 ± 10 hours) than that in the heparin-treated group (50 ± 21 hours; P value < 0.001). Local hemorrhage at puncture sites (two cases; managed after SK discontinuation), local packing in one case and suturing in another were recorded as complications in the SK group. Also, in the SK-treated group, one patient developed seizure and hemiparesis with no evidence of CNS hemorrhage on brain computed tomography. In the heparin-treated group, in one infant, epistaxis was observed post percutaneous transcatheter aortic commissuroplasty (PTAC), which disappeared after local packing and heparin discontinuation. Another patient developed seizure without CNS hemorrhage, which was controlled with conservative management.

## 5. Discussion

After cardiac catheterization in our 1788 patients, diminished/absent pulses in the lower extremity were immediately detected in 123 (6.7%) patients. Similar studies have previously reported various incidence rates. Ino et al. (10), Brus et al. (11) and Noori and Rezaii (7) reported post-cardiac catheterization femoral artery thrombosis incidence rates of 8.6%, 8.3%, and 7.3%, respectively, as opposed to Wessel et al. (12) and Bulbul et al. (9) who reported lower overall incidences of arterial complication of 3.6% and 5%, respectively, which are accounting for less than the findings of our study. These divergent results may be the consequence of different catheterization characteristics (especially in infants) or dissimilar systemic antithrombotic administrations. According to Freed et al. (8), lesser vascular trauma, appropriate inducer, suitable catheter type and size, regular washing, and heparinization during catheterization are factors capable of significantly reducing the possibility of vascular thrombosis during or after catheterization. Our results indicated that, after immediate heparin therapy, in accordance with the recommendations of the American College of Chest Physicians (13) for patients with impaired arterial circulation, pulses returned to the normal range in more than half of the study population. There are several studies confirming the efficacy of heparin in the treatment of arterial complications (10, 11).

The main finding of the present study was that SK (2000 U/kg loading dose, followed by 1000 U/kg/h by continuous infusion) exhibited a more reliable antithrombolytic effect for the removal of femoral artery thrombosis than did heparin in our pediatric patients. After SK therapy, normal pulse of the lower extremity was palpable in 88.2% of the patients: this seems a desirable outcome in comparison with similar studies. Wessel et al. (12) demonstrated that after SK infusion, pulses and systolic blood pressure returned to the normal ranges in the absence of any serious complications in 88% of the cases. Brus et al. (11) observed that in patients with femoral artery thrombosis, arterial perfusion was normalized in 89% of the SK-treated infants and children, although, in some patients, they increased the dose of SK after another loading dose.

The mechanism of action of SK has previously been documented. A protein produced by group C beta-hemolytic streptococci, SK is a fibrin-dependent plasminogen activator agent, which converts plasminogen to plasmin and lyses insoluble fibrin (14). In different investigations, SK has been administered in various doses with a view to successfully resolving femoral artery complications. Brus et al. (11) used different loading and infusion doses of SK (similar to the dose in our study) in nine pediatric patients and observed groin hematoma, despite successful treatment, in a child, after 54 h of SK therapy. Ali et al. (15) administered SK in a bolus dose of 5000 U/kg, followed by a maintenance dose of 2000 U/kg/h for 2 - 3 days, and managed to treat vascular thrombosis in three of the four children in their study. There is also a report of the effective and safe treatment of aortic thrombosis with an intravenous infusion of low-dose (1000 U/kg) SK after a mean interval of 2.2 days (16). Treatment with SK is occasionally associated with some complications, the most notable of which is bleeding (11). In our study, post-procedural complications occurred in only five patients: minor bleeding at puncture site in two, local packing in one, suturing in one and seizure and hemiparesis in one, all of which were successfully managed and there were no major/mucosal bleeding or other critical complications. The results of our study, with respect to the complication rates, indicate that we made use of SK in appropriate doses for the treatment of femoral artery thrombosis.

The occurrence of post-catheterization femoral artery thrombosis is more prevalent in young children and is occasionally associated with procedure-related characteristics. Immediate heparin therapy is a useful modality in the treatment of arterial complications in children, which underscores the significance of post-procedural lower-extremity assessment via physical examination or Doppler ultrasound. Furthermore, in children with femoral artery thrombosis after cardiac catheterization, thrombolytic therapy with SK in appropriate doses is a safe and effective treatment. The inherent risk in the cardiac catheterization of heparin-treated infants and young children requires supplementary attention not only to the selection of appropriate sheaths during cannulation, but also to the assessment of activated clotting time (ACT). Moreover, successful management of post-procedural bleeding requires well-trained staff in tandem with comprehensive assessment of the patient and meticulous selection of the interventional modality. Our results confirm the efficacy of thrombolytic therapy in the treatment of artery complications following cardiac catheterization. Notwithstanding thrombolytic therapy, however, pulse disturbances were detected in a small number of our pediatric patients. The affected leg in these children was viable, which suggests the formation of collateral vessels to maintain oxygenation in the tissue/organ. Longer follow-up of these patients is necessary to shed sufficient light on this issue.
